# Public health approach to hearing across the life course: a call-for-papers 

**DOI:** 10.2471/BLT.18.221697

**Published:** 2018-09-01

**Authors:** Shelly Chadha, Alarcos Cieza, Karen Reyes

**Affiliations:** a Management of Noncommunicable Diseases, Disability, Violence and Injury Prevention, World Health Organization, avenue Appia 20, 1211 Geneva 27, Switzerland.

Hearing is important for achieving a good quality of life, because a partial or total inability to hear is a barrier to communication and language development. Hearing loss, especially disabling hearing loss, is associated with delayed cognitive development in children and early cognitive decline in older adults.^1^ Disabling hearing loss refers to hearing loss greater than 40 dB in the better hearing ear in adults and greater than 30 dB in the better hearing ear in children.^2^ Unless such hearing loss is addressed in a timely manner, it has a profound impact on those affected as well as on their families and communities. Unaddressed hearing loss restricts social integration and educational and employment opportunities, hampers emotional well-being and poses an economic challenge at both the individual and national levels.^3^ Many of the causes that lead to hearing loss are preventable. WHO estimates that 60% of hearing loss occuring in childhood can be prevented through public health strategies including immunizations, better neonatal care, reduced environmental noise, decreased use of ototoxic medications and effective screening for ear infections.^4^ People of all ages with hearing loss can benefit greatly from timely, appropriate and cost-effective interventions, such as speech therapy and hearing devices.^5^

Unaddressed hearing loss is one of the leading causes of morbidity; it is ranked fourteenth in terms of disability adjusted life years and second in years lived with disability globally.^6^ Moreover, unaddressed hearing loss posed an annual cost of 750 billion United States dollars in 2015.^5^ Approximately 466 million people live with disabling hearing loss.^7^ The number of people with hearing loss is expected to rise in the coming decades, due mainly to changing population demographics, increasing exposure to risk factors such as noise, as well as persistence of untreated ear conditions such as otitis media.^8^ Most people with hearing loss reside in low- and middle-income countries, where hearing-care services are often lacking and the existing hearing care is not well integrated into health systems or accounted for in workforce strategies and health information management systems.

Hearing loss was highlighted at the World Health Assembly in 2017, when Member States unanimously adopted a resolution on the prevention of deafness and hearing loss.^9^ This resolution calls upon the World Health Organization (WHO) and its Member States to develop public health strategies to integrate ear and hearing care within the framework of the countries’ primary health-care systems. The aim of the resolution is that ear and hearing care should be accessible for all people, in line with the principles of universal health coverage. However, the implementation of this resolution and achievement of its mandate requires global public health action that has a strong evidence base and takes a life-course approach towards hearing. Such an approach targets various bio-psychosocial risk factors of hearing loss, such as ear infections, noise and vascular diseases, and interventions aimed at mitigating its adverse impact.^10^ The approach views the promotion of healthy hearing as a lifelong process that must be facilitated through public health action.^10^

Against this background, the *Bulletin of the World Health Organization* will publish a theme issue on the public health approach of hearing loss.

We welcome papers focusing on identifying and filling the gaps in evidence across comprehensive hearing-care services, from promotion of ear and hearing care, to screening, hearing devices and rehabilitation. In particular, the papers should report on unmet needs, outcomes of services, and effective and sustainable initiatives to reach underserved groups. Submission of papers reporting on both the magnitude of diseases and conditions, such as ear infections, meningitis and rubella, that can affect hearing, are encouraged. As well, papers addressing health system issues and promoting an intersectoral approach to ear and hearing care, such as looking beyond health. As much as possible, papers should seek to integrate examples from low- and middle-income countries across life course.

The deadline for submission is 30 November 2018. Manuscripts should be submitted in accordance with the *Bulletin’s *guidelines for contributors (available at: http://www.who.int/bulletin/volumes/96/1/18-990118/en/), and the cover letter should mention this call for papers.
